# Advances in Microsecond Time-Resolved Cryo-EM

**DOI:** 10.1063/4.0000815

**Published:** 2025-10-27

**Authors:** Ulrich J. Lorenz

**Affiliations:** EPFL, Lausanne, VD, Switzerland

## Abstract

Protein structure determination and prediction have made stunning progress. In contrast, it is generally not possible to observe proteins as they perform their tasks, which leaves our understanding of these nanoscale machines fundamentally incomplete. My group has recently introduced a novel approach to time-resolved cryo-EM that improves its time resolution by about 3 orders of magnitude, making it fast enough to observe the microsecond dynamics of proteins that are frequently associated with function. Our method involves melting a cryo sample with a laser beam, which allows dynamics of the embedded particles to occur in liquid if a suitable stimulus is provided. When the heating laser is switched off, the sample rapidly revitrifies, trapping the particles in their transient configurations, in which we can subsequently image them. As I will illustrate, this makes it possible to watch protein dynamics that were previously unobservable. Our method therefore promises to fundamentally advance our understanding of protein function. We also demonstrate that laser melting and revitrification of cryo samples can be used to overcome preferred particle orientation, an issue that still causes many cryo-EM projects to fail. Finally, I will show a new approach that significantly expands the utility of our method by extending its temporal observation window. This allows us to follow the structural evolution of conformational ensembles in a temperature jump experiment on a timescale of hundreds of microseconds.

